# Real-World Patterns of Delayed Cutaneous Adverse Reactions to Antimicrobial Therapy in Children: A Case Series and FAERS Analysis Across Age Groups

**DOI:** 10.3390/antibiotics15080749

**Published:** 2026-08-03

**Authors:** Vera Battini, Martina Loiodice, Giulia Mosini, Stefania Cheli, Ilaria Mariani, Sara Dal Molin, Sofia Dinegro, Gianvincenzo Zuccotti, Emilio Clementi, Sonia Radice, Valentina Fabiano, Carla Carnovale

**Affiliations:** 1 Pharmacovigilance & Clinical Research, International Centre for Pesticides and Health Risk Prevention, ASST Fatebenefratelli-Sacco, Department of Biomedical and Clinical Sciences, Università degli Studi di Milano, 20157 Milan, Italy; vera.battini@unimi.it (V.B.); giulia.mosini@asst-fbf-sacco.it (G.M.); stefania.cheli@asst-fbf-sacco.it (S.C.); ilaria.mariani@unimi.it (I.M.); dalmolin.sara@asst-fbf-sacco.it (S.D.M.); dinegro.sofia@asst-fbf-sacco.it (S.D.); emilio.clementi@unimi.it (E.C.); sonia.radice@unimi.it (S.R.); 2 Unit of Pediatrics, Department of Biomedical and Clinical Sciences, “Vittore Buzzi” Children’s University Hospital, Università degli Studi di Milano, 20154 Milan, Italy; martina.loiodice@unimi.it (M.L.); gianvincenzo.zuccotti@unimi.it (G.Z.); valentina.fabiano@unimi.it (V.F.); 3 Scientific Institute IRCCS E. Medea, Bosisio Parini, 23842 Lecco, Italy

**Keywords:** adverse drug reactions, skin reactions, children, antimicrobial therapy, pharmacovigilance

## Abstract

**Background/Objectives**: Cutaneous adverse drug reactions (CADRs) account for approximately 45% of all adverse drug reactions. Although most are self-limiting, some may progress to severe cutaneous adverse reactions (SCARs). In children, delayed rashes associated with antimicrobial therapy, particularly beta-lactams, are often misclassified as drug allergies, leading to unnecessary antibiotic avoidance and potentially suboptimal antimicrobial prescribing. This study aimed to characterize delayed antimicrobial-associated CADRs in children and to investigate reporting patterns and factors associated with their clinical management across age groups. **Methods**: Real-world data from pediatric patients hospitalized in 2023 at the “Ospedale dei Bambini Vittore Buzzi” (Milan, Italy) were integrated with Individual Case Safety Reports from the FDA Adverse Event Reporting System (FAERS). Only cases with documented treatment durations were included. Clinical characteristics, antimicrobial exposure patterns, and factors associated with the reporting of delayed rashes were evaluated. **Results**: Five pediatric patients developed delayed CADRs after 19–22 days of antimicrobial therapy. Infectious and immunological investigations were negative, and symptoms resolved following drug discontinuation. FAERS analysis identified 97 delayed rash reports, associated with prolonged treatment duration, frequent polytherapy, and higher reporting rates for vancomycin, teicoplanin, and beta-lactam combination regimens. Logistic regression showed that age and polypharmacy were significantly associated with reporting patterns of delayed rash and therapy continuation among reported cases. **Conclusions**: Among reported cases, delayed antimicrobial-associated CADRs were associated with age and polypharmacy. Improved recognition of these reactions may facilitate appropriate clinical management, support more informed prescribing decisions, and reduce inappropriate antibiotic allergy labeling. Further studies are needed to validate these findings and refine risk-based management strategies in pediatric patients.

## 1. Introduction

The skin is the most affected organ in adverse drug reactions (ADRs), accounting for ~45% of all cases. The reported prevalence of cutaneous ADRs (CADRs) is approximately 1–3% among adult patients and 2.5% among pediatric patients receiving drug treatment, although these estimates refer to drug-treated populations without a specifically defined healthcare setting [1]. While typically self-limiting, 2–6.7% of CADRs may progress to severe cutaneous adverse reactions (SCARs), which have a global incidence of 0.4–1.2 cases per million annually, varying by population and the drug involved [2].

CADRs are classified as immediate or delayed based on onset. Immediate reactions, occurring within minutes to hours, include urticaria, angioedema, rhinitis, bronchospasm, and anaphylaxis, and are mediated by drug-specific IgE via type I hypersensitivity [3]. In contrast, delayed drug reactions develop over days to weeks and may present as maculopapular exanthems, morbilliform eruptions, urticarial-like lesions, fixed drug eruptions, or, less frequently, severe cutaneous adverse reactions (SCARs) such as DRESS, AGEP, Stevens–Johnson syndrome (SJS), and toxic epidermal necrolysis (TEN). Their immunopathogenesis is heterogeneous and not fully elucidated [4]. Most non-immediate CADRs are T-cell-mediated and are classified as type IV hypersensitivity reactions. Allergic maculopapular exanthems, for example, are associated with drug-specific CD4+ T cells that induce keratinocyte apoptosis through a perforin-dependent mechanism following MHC class II presentation [1].

Antibiotics are the most common triggers of immune-mediated drug reactions, ranging from mild hypersensitivity to severe organ toxicity and SCARs [5]. Among them, beta-lactams, particularly amoxicillin alone or combined with clavulanic acid, are the drugs most frequently implicated, accounting for approximately 15% of all drug-induced skin reactions and 55% of antibiotic-related cases [6,7]. In pediatric patients, the prevalence of CADRs may reach 12% [8]. However, many non-immediate eruptions, especially maculopapular exanthems, are associated with underlying infections rather than true drug hypersensitivity, making their diagnosis particularly challenging [9,10]. Accurate recognition of delayed CADRs is therefore essential to avoid inappropriate beta-lactam allergy labeling, which may unnecessarily restrict the use of first-line antibiotics and compromise optimal antimicrobial prescribing. In this context, pediatric antimicrobial stewardship programs increasingly emphasize appropriate diagnostic assessment, multidisciplinary clinical evaluation, and optimization of antibiotic prescribing as key strategies to improve prescribing practices while minimizing unnecessary antimicrobial exposure and overprescribing [11,12].

Despite recent advances, data on the clinical characteristics of delayed CADRs in pediatric patients undergoing prolonged intravenous antibiotic therapy—particularly for osteoarticular and soft tissue infections—remain limited. Information on onset, risk factors, and relevant features is scarce, hindering clinical decision-making.

Real-world data (RWD) and real-world evidence (RWE) are essential to enhance diagnostic accuracy and inform therapeutic strategies, ultimately improving patient management.

In this context, we conducted a pharmacoepidemiological study combining a pediatric case series from the Ospedale dei Bambini “Vittore Buzzi” in Milan, Italy, with an analysis of the FDA Adverse Event Reporting System (FAERS), one of the largest pharmacovigilance databases supporting post-marketing drug safety surveillance. This combined real-world approach aimed to characterize delayed antimicrobial-associated cutaneous adverse drug reactions (CADRs) in children, compare reporting patterns across pediatric, adult, and elderly populations, and investigate factors associated with their reporting and clinical management.

## 2. Results

### 2.1. Clinical Features of Patients with Delayed Antimicrobial-Associated Cutaneous Reactions

Five pediatric patients (three females and two males; mean age 4.8 years) developed delayed CADRs during hospitalization in the year 2023 for severe bacterial infections. Their main clinical characteristics are summarized in Table 1.

The reactions occurred after prolonged antimicrobial exposure, with symptom onset being between days 19 and 22 of therapy. Three patients were receiving treatment for acute osteomyelitis, one for a paravertebral retropharyngeal abscess, and one for suppurative submandibular lymphadenitis. Four patients were exposed to beta-lactam-based regimens from the start of therapy (ampicillin–sulbactam or oxacillin), whereas one patient was at first receiving intravenous vancomycin and received ampicillin–sulbactam later as an added therapy.

Clinical manifestations included erythematous maculopapular, morbilliform, vasculitic-like, and urticarial eruptions. Fever was present in three cases, while mucosal involvement was not observed. Two patients developed palmoplantar maculopapular eruptions during prolonged ampicillin–sulbactam therapy. The patient receiving vancomycin developed a morbilliform rash after 22 days of treatment, despite extensive investigations excluding alternative infectious or immunological causes. The remaining two patients experienced pruritic eruptions during treatment with beta-lactam-containing regimens: one associated with vasculitic features and fever and the other presented as an urticarial-like eruption.

Comprehensive microbiological and immunological investigations were performed in all cases to exclude alternative etiologies. Blood cultures, respiratory viral testing using multiplex viral respiratory panels and serological investigations for most common viruses and bacteria that may cause cutaneous eruptions (Cytomegalovirus, Epstein–Barr virus, Adenovirus, Coxsackievirus, Parvovirus B19, Mycoplasma pneumoniae, and Chlamydia pneumoniae) were negative when performed. Specific IgE testing for penicillin and amoxicillin was also negative in the evaluated patients. Although one patient tested positive for Influenza A, the temporal relationship and clinical presentation suggested that the viral infection was unlikely to account for the cutaneous manifestations.

Antimicrobial therapy was discontinued at symptom onset in all patients, leading to complete resolution of the reactions. In two cases, we performed a switch to another antibiotic therapy (in case 1 we switched to clindamycin and in case 3 we switched to teicoplanin); in the other three cases, we did not prescribe any alternative antibiotic therapy. In two cases (case 2 and 3), the occurrence of the reactions led to a significant prolongation of hospital stay (1 week more in case 2 and 1 month more in case 3). All cases were reported to the hospital pharmacovigilance service. Collectively, based on the temporal relationship, resolution after withdrawal of the suspected antimicrobial(s), exclusion of alternative infectious and immunological causes, and assessment according to the Naranjo Adverse Drug Reaction Probability Scale, all five cases were classified as having a probable causal association with the suspected antimicrobial(s).

### 2.2. Characterization of Delayed Antimicrobial-Associated Cutaneous Reactions in the FAERS

To further contextualize the findings from the hospital cohort, a pharmacovigilance analysis was performed using the FAERS database.

Within the 15,328,577 reports recorded in the database during the study period, 10,594,628 reports were excluded during the data cleaning procedure, and 4,731,673 were excluded for not reporting rashes after the use of the drugs of interest for which we have information about treatment duration. Thus, 258 pediatric, 1047 adult, and 971 elderly reports met the inclusion criteria. Among these, all adult (n = 587) and elderly (n = 481) reports not included in the cohort analyses were classified as rapid rash cases (rash occurring within the first 7 days of therapy). To fulfill the aims of our study, we included 97 pediatric, 460 adult, and 490 elderly reports with delayed rashes, together with 161 pediatric reports with rapid rashes for comparison (Figure S1). Descriptive analysis of demographic and therapeutic characteristics is reported in Table 2.

In most cases, the reporters were physicians (48%). When comparing pediatric patients with delayed rash versus those with rapid rash, younger children were more frequently reported in the rapid rash group, with a median age of 3 years vs. 7 years in the delayed group (*p* < 0.05) and there were no differences regarding sex (*p* = 0.42). The median (25th–75th percentiles) therapy duration was 13 (9–26) days for the delayed group and 2 (1–4) days for the rapid group. The median (25th–75th percentiles) time to onset reported similar values [12 (9–24) vs. 1 (1–4) days, respectively], since most of the patients withdrew from therapy at the occurrence of the events, with a slightly yet significant higher percentage in the rapid group (72% of cases in the delayed group vs. 86% in the rapid group, *p* < 0.05). The use of Vancomycin/Teicoplanin and polytherapy with more than one beta-lactam drug or more than one drug of interest were significantly higher in the delayed group compared to the rapid group (*p* < 0.05). Concomitant use of ATC class J01E (sulfonamides and trimethoprim) and J01G (aminoglycoside antibacterials) were also more frequent in the delayed group (*p* < 0.05). When comparing pediatric patients with delayed rash to the other age groups, the median (25th–75th Percentiles) time to onset that was reported in children was earlier than in adults and older patients [14 (10–22) and 19 (11–27) days, respectively], with a significant difference between the elderly people compared with the other two groups (*p* < 0.05 for both adults vs. elderly people and children vs. elderly people, Dunn’s Test). Pediatric cases had significantly higher percentages of treatment discontinuation at the occurrence of the events (72%, *p* < 0.05). The use of more than one drug of interest was highest in elderly patients, followed by adults and children (79%, 75%, 67%, respectively, *p* < 0.05). The concomitant use of ATC class J01G (aminoglycoside antibacterials) was significantly higher in children (18%, *p* < 0.05), while concomitant use of ATC class J01E (sulfonamides and trimethoprim), the use of Vancomycin/Teicoplanin, and polytherapy with more than one beta-lactam were not significantly different among age classes when considering delayed rash (*p* = 0.68, *p* = 0.63 and *p* = 0.18, respectively). The indication therapy related to infections that were retrieved in the individual case safety reports (ICSRs), when reported, are shown in Figure S2: the most common reported indications for antibiotic use were infections not elsewhere classified (NEC) and ear infections across all age groups.

**Figure 1:**
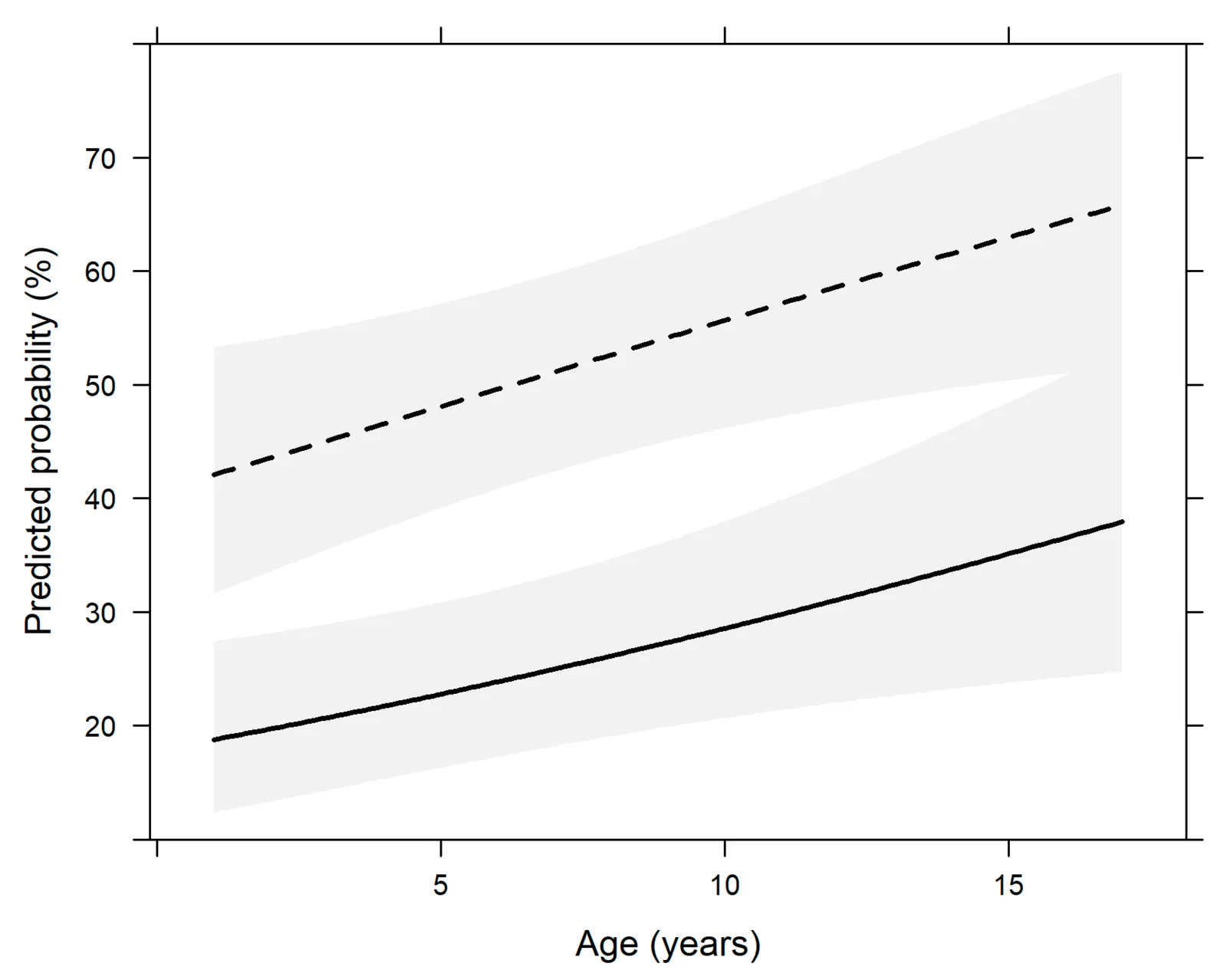
Adjusted predicted probability (%) of delayed rash in pediatric patients across age, stratified by exposure to a single antibiotic versus multiple antibiotics of interest. Predictions were derived from a multivariable logistic regression model including age (in years) and antibiotic exposure (single versus multiple antibiotics of interest). Probabilities represent adjusted estimates obtained from the inverse logit transformation of the linear predictor. Curves are stratified by antibiotic exposure to illustrate differences in predicted risk between groups, while accounting for covariates included in the model. The solid line represents exposure to a single antibiotic of interest, while the dashed line represents exposure to multiple antibiotics of interest. Shaded areas indicate 95% confidence intervals.

In the FAERS dataset, increasing age was associated with higher odds of reporting delayed rather than rapid rash among pediatric patients [OR (95% CI) = 1.95 (1.16–3.28)]. The presence of multiple drugs of interest in the report was also independently associated with higher odds of delayed rash [OR (95% CI) = 3.14 (1.84–5.37)]. After adjustment for antibiotic exposure, reports of older children showed a higher predicted probability of delayed rash compared with younger patients. This association is illustrated in Figure 1, which shows adjusted predicted probabilities (%) derived from the logistic regression model across age, stratified by antibiotic exposure (monotherapy or polytherapy with antibiotics of interest).

**Figure 2:**
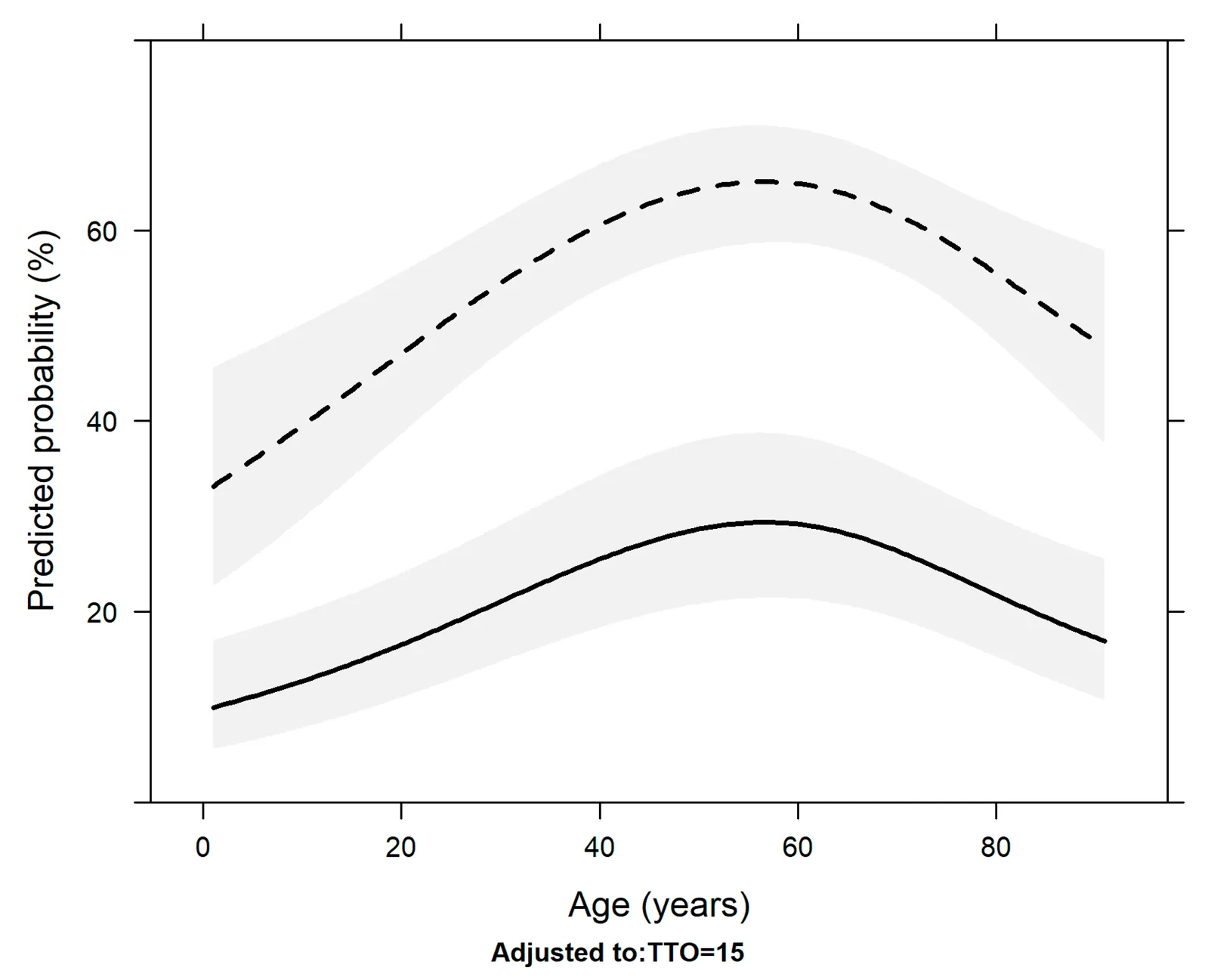
Adjusted predicted probability (%) of antibiotic discontinuation following delayed rash across age in pediatric patients. Predictions were obtained from a multivariable logistic regression model including age (in years), time-to-onset (TTO, in days), and antibiotic exposure (single versus multiple antibiotics of interest). Probabilities represent adjusted estimates derived from the inverse logit transformation of the linear predictor. Time-to-onset was fixed at its median value (15 days) and curves are stratified by antibiotic exposure to illustrate adjusted differences between groups. The solid line represents exposure to a single antibiotic of interest, while the dashed line represents exposure to multiple antibiotics of interest. Shaded areas indicate 95% confidence intervals.

Among reports of delayed rash, age was significantly associated with treatment discontinuation, with evidence of a non-linear relationship as modeled using restricted cubic splines. Exposure to more than one antibiotic of interest was independently associated with substantially higher odds of treatment discontinuation [OR (95% CI) = 4.50 (3.13–6.47)]. Longer time to onset (TTO) was also associated with higher odds of treatment discontinuation [OR (95% CI) = 1.46 (1.13–1.88)]. To facilitate interpretation of the non-linear effect of age, adjusted predicted probabilities (%) of treatment discontinuation were estimated across the age range while fixing TTO at its median value (15 days) and stratifying by antibiotic exposure (monotherapy versus polytherapy with antibiotics of interest). As shown in Figure 2, the predicted probability of treatment discontinuation increased with age until approximately the sixth decade of life and then gradually declined, with consistently higher probabilities among reports involving polytherapy than monotherapy.

## 3. Discussion

Cutaneous adverse drug reactions are frequently observed across all age groups. While rapidly presenting cutaneous reactions are more often caused by allergy, delayed-onset skin reactions pose a greater diagnostic challenge, and a better comprehension of factors involved in their occurrence may help physicians in improving their management and in making wiser decisions that could be based on better consideration of the risk/benefit ratio. Our analysis of the FAERS database aimed to characterize reporting patterns of delayed CADRs by comparing pediatric, adult, and elderly reported cases. Antimicrobials, particularly beta-lactams, are among the most frequently implicated drugs in delayed CADRs in children, whereas in adults and older patients, a broader spectrum of medications, including cardiovascular, neurological and anti-inflammatory agents, may contribute because of greater multimorbidity and polypharmacy. In our pediatric case series, beta-lactam-based regimens accounted for most delayed CADRs, consistent with the well-recognized role of beta-lactam antibiotics as the leading cause of suspected antibacterial hypersensitivity reactions in children [5,9,10].

When comparing pediatric patients with antibiotic-associated delayed rashes versus those with rapid rashes, younger children were more frequently represented in the rapid rash group, with a significantly lower median age (3 vs. 7 years, *p* < 0.05). This finding suggests that, in the first years of life, rapid rashes are more frequently reported. This may be related to greater clinical attention to potential allergic mechanisms in this age group, where antibiotic exposure is frequent and often closely monitored. In addition, clinicians may be more likely to document even mild cutaneous reactions in younger patients.

Across all analyses in the FAERS, exposure to more than one antibiotic of interest was consistently associated with higher adjusted probabilities of adverse outcomes. In reports involving pediatric patients, polytherapy was associated with an increased predicted probability of delayed rash, and the same exposure was also strongly associated with higher predicted probability of treatment discontinuation among cases with delayed reactions. The consistency of this association across different outcomes suggests that antibiotic polytherapy may represent an independent and robust factor associated with both the occurrence and clinical management of cutaneous adverse drug reactions. Importantly, the parallel separation of the predicted probability curves across exposure groups further supports the stability of this effect across the age spectrum.

Among pediatric reports, concomitant exposure to more than one drug of interest was significantly more frequent in delayed than rapid rash cases (67% vs. 39%, *p* < 0.05), and logistic regression confirmed a strong association between polytherapy and delayed rash reporting (OR 3.14, 95% CI 1.84–5.37).

Polypharmacy is considered an important contributor to the risk of cutaneous adverse drug reactions (CADRs), particularly in older adults, as it increases exposure to potential culprit drugs and the likelihood of drug–drug interactions. In this population, multimorbidity, age-related pharmacokinetic and pharmacodynamic changes, and polypharmacy further increase both the risk and the clinical severity of CADRs. Therefore, age-related differences in drug utilization and comorbidity profiles should be considered when interpreting reporting patterns across different age groups [13,14,15,16]. These factors may also contribute to the greater clinical severity of CADRs reported in older patients.

In the descriptive analysis of FAERS cases with delayed rash across all ages, treatment discontinuation more frequently appeared in children compared with other age groups, whereas in adults and elderly patients’ therapy was more often continued after rash onset. However, looking at the multivariable analysis, which accounted for age (modeled using restricted cubic splines), time-to-onset, and antibiotic exposure, these crude differences should be interpreted with caution. The adjusted models highlight that the association between age and treatment discontinuation is non-linear across the age spectrum. These differences between crude proportions and adjusted predictions underline the importance of accounting for non-linear effects and clinical covariates when interpreting these associations. Overall, age appeared to influence treatment discontinuation patterns in a non-linear fashion after adjustment for confounders.

Nevertheless, when focusing on pediatric patients alone, the proportion of treatment discontinuation at rash onset was lower in the delayed rash group compared with the rapid rash group (72% vs. 86%, *p* < 0.05). This suggests a tendency toward continuation of therapy in delayed reactions, although the magnitude of this difference appears limited. This pattern may be consistent with a more cautious approach to antibiotic discontinuation in cases where immediate hypersensitivity is considered less likely, as well as with the clinical need to balance the risks of interruption of an ongoing therapy against the risk of adverse outcomes. These findings underscore the importance of careful, individualized clinical assessment of delayed CADRs. Accurate recognition of these reactions may facilitate informed clinical decision-making regarding the continuation, modification, or discontinuation of antimicrobial therapy. Such decisions may benefit from a multidisciplinary approach involving infectious disease specialists, allergists, clinical pharmacists, and treating physicians, particularly in complex cases where the risks and benefits of continued antimicrobial treatment need to be carefully balanced.

The multivariable analysis of FAERS cases further showed that continuation of therapy was less likely at the extremes of the age distribution. This finding may reflect differences in clinical decision-making for very young and older patients, who are generally considered more vulnerable to adverse drug reactions because of age-related physiological characteristics and, in older adults, multimorbidity and polypharmacy [17,18]. However, this interpretation remains speculative, as the FAERS database does not provide direct information on the clinical severity of individual cases.

In our pediatric case series, all reactions occurred after prolonged antimicrobial exposure (19–22 days), and complete resolution followed antimicrobial discontinuation in every patient; consistent with the pharmacovigilance findings, our clinical team tended to discontinue the ongoing antibiotic therapy, a decision further justified by the fact that in all cases, the reactions occurred during prolonged treatment courses that were nearing completion and therefore, the risks outweighed the benefits. The clinical phenotypes observed in our patients, including maculopapular, morbilliform, urticarial-like and vasculitic-like eruptions, are representative of the heterogeneous presentation of delayed CADRs. Unfortunately, the level of clinical detail available in FAERS does not allow for a reliable characterization of delayed CADR phenotypes across age groups.

Future prospective real-world studies incorporating standardized clinical causality assessment will be essential to better define the etiology of delayed CADRs and to complement the hypothesis-generating evidence provided by spontaneous reporting systems.

### Strengths and Limitations

ICSR databases offer a unique perspective on drug-related adverse events by capturing a broad spectrum of patient demographics, comorbidities, and concomitant medications encountered in routine clinical practice, thereby complementing the evidence generated from randomized clinical trials [19,20]. The FAERS, mainly representative of the United States, also gathers serious adverse events from the rest of the world, providing a global perspective.

Cases reporting events that occurred >1 days after the end of the therapy were excluded, since the reason behind the associations between the antimicrobial therapy and the occurrence of the event should be explored in detail, as pharmacokinetic and pharmacodynamic aspects must be considered, especially considering concomitant therapies [21].

We acknowledge the limitations of this study, which are inherent to the nature of spontaneous reporting systems (under-reporting and possible selective reporting) as well as specifically related to the study design. In addition, the pediatric case series included a limited number of patients, which restricts the generalizability of the clinical observations. Given the lack of a denominator, namely subjects exposed to the drug(s), and the expected under-reporting phenomenon, disproportionality measures and their magnitude cannot quantify the real risk in clinical practice but can only offer a suggestion about an increased risk of adverse event reporting and not of adverse event occurrence. Consequently, incidence cannot be calculated [20].

Our FAERS analysis also showed that, in most cases, the reporters were physicians. This result is likely, since we selected highly specific terms for “rash”, which would probably have captured a higher proportion of consumer reports. In addition, our inclusion criteria required complete information on both treatment start and end dates, resulting in a highly curated dataset with a high degree of completeness. Although this level of detail is not always necessary for routine pharmacovigilance analyses, it was essential to address our specific research question [20].

Moreover, verification of events through clinical features, including laboratory and instrumental tests, comorbidities and adjustment of therapeutic regimens is limited due to missing data and no access to narratives. Consequently, reliable characterization of delayed CADR phenotypes in the FAERS dataset was not possible. In addition, differences in multimorbidity, polypharmacy, and prescribing patterns across age groups may influence both the occurrence and reporting of adverse drug reactions and should therefore be considered when interpreting age-related comparisons.

Several additional factors may result in selective reporting and relevant ability to detect disproportionality, including known and largely reported drug–event combinations (the so-called competition bias); the setting, pattern, and extent of use (which are related to marketing life and evolving guidelines), as well as the attitude of clinicians toward reporting. Therefore, channeling bias cannot be ruled out [20].

To improve data quality and reduce potential confounding, we applied a rigorous data-cleaning process, removed duplicate reports, retained only reports with sufficient information to estimate treatment duration and time-to-onset, and excluded events occurring more than one day after antimicrobial discontinuation. Nevertheless, residual confounding and reporting bias are inherent to spontaneous reporting systems and cannot be eliminated. Therefore, the identified factors should be interpreted as associations with reporting patterns among reported cases rather than as causal risk factors or predictors of delayed CADRs.

## 4. Materials and Methods

### 4.1. Clinical Evaluation of Delayed Antimicrobial-Associated Cutaneous Reactions

We retrospectively analyzed clinical records of all patients who were hospitalized in 2023, in the Pediatric Department of “Ospedale dei Bambini Vittore Buzzi” in Milan, for treatment of severe infections, requiring prolonged (at least 14 days) intravenous antibiotic therapy, and developing delayed CADRs at least 7 days after the initiation of the suspected causative antibiotic. We found a total of five patients meeting the inclusion criteria and all were included in the analysis.

Patients’ characteristics and treatment details were retrieved from paper and electronic patients’ records. We anonymously collected data about patients’ demographic and principal diagnosis (including clinical features, imaging and microbiological investigations). We also collected data about antibiotic treatment, time of initiation, dosages, and eventually associated or concomitant therapies. For characterizations of CARDs, we considered the clinical features of the reactions, time of onset, actions required to manage their occurrence (including eventual discontinuation of suspected drug, dosage modifications, and treatment of ADRs) and to better characterize them (for example, dermatological evaluation, microbiological investigations to rule out concomitant infections, and IgE testing). For the five pediatric cases, causality was retrospectively assessed using the Naranjo Adverse Drug Reaction Probability [22].

All collected data were retained in a password-protected Excel database. Written informed consent was obtained from the patients’ parents or legal guardians at the time of hospitalization, as per local routine practice.

### 4.2. Pharmacovigilance Analysis of the FAERS

#### 4.2.1. Data Source

The database used for this analysis is the FAERS, a publicly available spontaneous reporting system containing anonymized spontaneous reports of adverse events that are submitted to the FDA by consumers, healthcare professionals and pharmaceutical industries. The study period (2015Q1–2024Q2) was chosen for this study. The data was already anonymized, thus complying with privacy regulations and ensuring ethical data usage. Adverse events in FAERS are classified using MedDRA (version 24.0) at the Preferred Terms level, with drug entries recorded as free text by reporters, encompassing prescription medications, over-the-counter drugs, biologics, and advanced therapies. Free-text drug names were standardized using the Open-Source Living DiAna Dictionary [23], ensuring consistent coding and classification of drugs. When multiple versions of a case were available, only the last one was retained.

Duplicate entries were identified and removed through checking to increase the probability that each adverse event report was unique. Values in the fields of sex, event date, age, country of occurrence, and drugs were considered appropriate for duplication; therefore, reports lacking this essential information were excluded from the analysis to ensure data quality. These data pre-processing steps were implemented to enhance data accuracy and reliability, ensuring the integrity of the subsequent results. From this cleaned dataset, the cohort study was then derived.

#### 4.2.2. Study Population and Study Design

The study population comprises only ICSRs reported in the FAERS database for which it was possible to determine the duration of therapy for the suspected antimicrobial therapy (ATC code: J01C-beta-lactam antibacterials, penicillin, J01D-other beta-lactam antibacterials, J01XA01/J01AX02-Vancomycin/Teicoplanin). If the start date of the therapy was available but the end date was missing, the date of the adverse event was assumed as being the end date of therapy. Time to onset was calculated as the time that occurred between the first administration of the drug of interest and the event date. For the retrieval of the events of interest, a list of clinically relevant MedDRA terms was used (see Supplementary Material S1).

**Figure 3:**
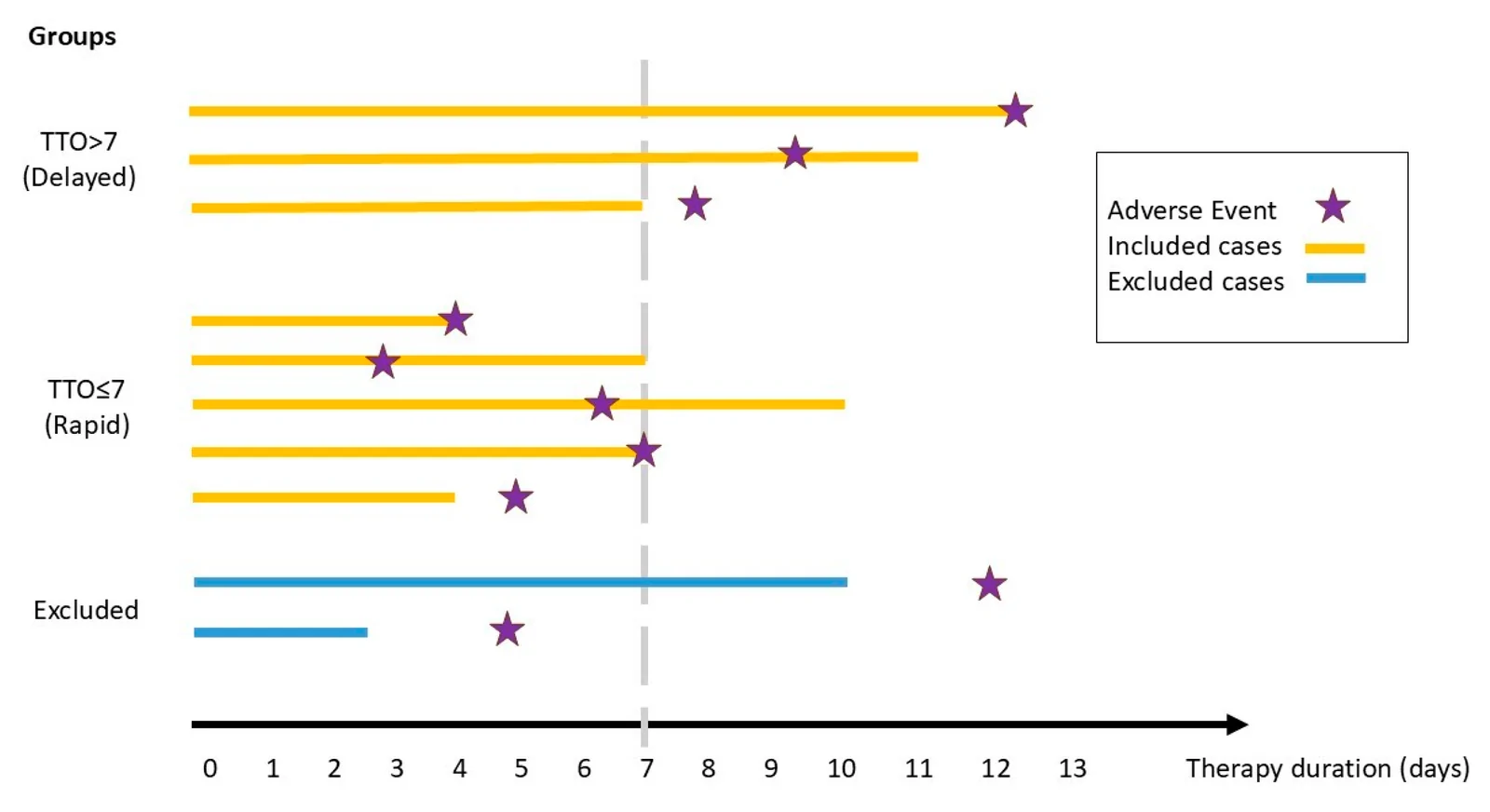
Pharmacovigilance study design. The diagram reports the classification of cases according to type of CADR onset and treatment duration. Yellow horizontal bars represent included cases, stratified into rapid-onset rash (TTO ≤ 7 days) and delayed-onset rash (TTO > 7 days). Blue horizontal bars represent excluded cases, i.e., reports where the event occurred >1 days after the end of the therapy. Purple stars indicate the occurrence of the adverse event within each subgroup. The vertical dashed line at day 7 represents the predefined threshold used to distinguish between rapid and delayed onset reactions. The *x*-axis indicates therapy duration in days.

As described in Figure 3, to increase the comparability between the FAERS population and our pediatric case series, and to minimize uncertainty regarding drug-event temporal association in spontaneous reports, only events occurring during therapy or within one day after treatment discontinuation were included; therefore, as described in Figure 1, events that occurred >1 days after the end of the therapy were excluded, and the population was divided into the following groups: patients who developed a rash within the first 7 days of therapy, referred to as the “rapid” group, and patients who developed a rash after the first 7 days of therapy, referred to as the “delayed” group. Since our focus was on delayed adverse reactions, two comparisons were then performed within specific subgroups to identify potential effect modifiers and assess whether the association between antimicrobial therapy and rash varies across different patient populations: (1) pediatric patients in the delayed group compared with pediatric patients in the rapid group, and (2) pediatric patients (<18 years) in the delayed group compared with adults (18–64 years) and elder patients (>64 years) in the delayed group.

The primary analysis consisted of exploring the patients’ demographic and therapeutic characteristics and duration dependency; this allowed for a detailed examination of the association’s strength concerning drug exposure and the occurrence of the adverse event of interest. Subsequently, an exploratory analysis was performed to evaluate the probability within the pediatric reports of developing a delayed rash compared to rapid rash and the probability of continuing therapy after a delayed rash compared to a rapid discontinuation was also assessed across reports of all age groups.

#### 4.2.3. Statistical Analysis

The characteristics of cases exposed to antimicrobial therapy were described using the median and 25th–75th percentiles for continuous variables, with numbers and rates for categorical variables. Chi-square tests were employed to assess the association between categorical variables (Fisher’s test if expected values < 5%). The Mann–Whitney test for continuous variables was used for the comparison of the two pediatric groups, while the Kruskal–Wallis test with Pairwise Test for Multiple Comparisons of Mean Rank Sums (Dunn’s Test) was used when comparing the different age groups [Elderly (>64 years), Adults (18–64 years), Children (<18 years)] for subjects with delayed onset. A significance threshold of *p* < 0.05 was used to determine the statistical significance.

A primary multivariable logistic regression model was used to assess associations between clinical variables and delayed cutaneous adverse drug reactions (CARDs) in the pediatric population. The model included age (in years) and exposure to more than one antibiotic of interest as independent variables. Functional forms of continuous variables were assessed using restricted cubic splines. When no evidence of non-linear effects was observed, continuous variables were modeled as linear terms to improve model parsimony and interpretability. Accordingly, in the primary model age was included as a linear term. A second multivariable logistic regression model was used to assess associations with therapy discontinuation among patients of all ages experiencing delayed CARDs. This model included age (modeled using restricted cubic splines), time-to-onset (modeled using restricted cubic splines), and exposure to more than one antibiotic of interest. Results are reported as odds ratios with 95% confidence intervals (OR, 95% CI) and as adjusted predicted probabilities (%), derived using the inverse logit transformation of the linear predictor. Predicted probabilities were obtained by varying age while holding the remaining covariates at specified values. For the primary model, curves were stratified according to antibiotic exposure (single versus multiple antibiotics of interest). For the second model, TTO was fixed at its median value (15 days), and predicted probabilities were similarly stratified by antibiotic exposure. The regression analyses were exploratory in nature and aimed to estimate associations rather than develop or validate a predictive model.

Model estimates were obtained using the lrm function from the rms package in R software. All analyses were performed using R software, version 4.4.2 (R Foundation for Statistical Computing, Vienna, Austria).

## 5. Conclusions

By integrating hospital-based clinical observations with large-scale pharmacovigilance data, this study provides real-world evidence on delayed antimicrobial-associated CADRs occurring during prolonged antibiotic therapy. Across both datasets, age and antimicrobial polytherapy emerged as factors associated with delayed rash occurrence.

Although causality cannot be established from spontaneous reporting data, these findings highlight the importance of maintaining clinical vigilance for delayed CADRs during prolonged antimicrobial treatment, particularly in pediatric patients receiving combination regimens. Improved recognition of these reactions may help avoid inappropriate antibiotic allergy labeling and support more appropriate prescribing decisions when delayed CADRs are suspected. Moreover, awareness of delayed CADRs as a potential complication of prolonged antimicrobial exposure may facilitate a more balanced assessment of the risks and benefits of treatment continuation, especially when extended antibiotic courses are required for severe infections.

Further prospective studies are needed to validate these observations, clarify the underlying mechanisms, and define evidence-based approaches for risk stratification and management. Ultimately, timely recognition and appropriate management of delayed CADRs may represent an important component of antibiotic optimization, helping to reduce unnecessary treatment discontinuation while supporting safer and more individualized antimicrobial use in clinical practice.

## Figures and Tables

**Table 1:** Baseline demographic and clinical characteristics of patients with delayed antimicrobial-associated cutaneous reactions.

Id	Age, yrs	Sex	Weight (kg)	AB	Dose	Route of Administration	Diagnosis	Therapy Duration (Days)	TTO (Days)	AE	Concomitant Drugs
**1**	2	F	9.9	Ampicillin/ sulbactam	200 mg/kg/d	Intravenous	Chronic osteomyelitis	21	20	Erythematous maculopapular rash with fever	-
**2**	5	M	18.8	Ampicillin/ sulbactam	200 mg/kg/d	Intravenous	Chronic osteomyelitis	19	18	Erythematous maculopapular rash with fever	-
**3**	9	F	27	Vancomycin Ampicillin/ sulbactam	40 mg/kg/d 200 mg/kg/d	Intravenous	Osteomyelitis	22 22 for Van 8 for Ampi/sulb	22	Morbilliform rash with pruritus	Rifampicin Carvedilol Captopril Spironolactone
**4**	2	M	11.8	Ampicillin/ sulbactam	165 mg/kg/d	Intravenous	Parapharyngeal abscess	19	19	Diffuse maculo-pomphoid rash with vasculitic features and fever	Metronidazole
**5**	2	F	12	Ampicillin/ sulbactam Oxacillin	150 mg/kg/d 140 mg/kg/d	Intravenous	Cervical adenitis	12 9	21	Urticarial pruriginous rash	-

AB: antibiotic; AE: adverse event; d: die; F: female; M: male; tid: three times per die; TTO: time to onset.

**Table 2:** Demographic and therapeutic characteristics of the included cases collected in the FAERS.

Age Groups, Years	Pediatrics <18		*p* *	Adults 18–64	Elderly >64	*p* ^§^
TTO Groups	Delayed	Rapid		Delayed	Delayed	
n	97	161		460	490	
*Reporter type, n (%)*						
Consumers	10 (10)	31 (19)		29 (6)	31 (6)	
Healthcare professionals	10 (10)	24 (15)		46 (10)	45 (9)	
Medical doctors	52 (55)	73 (45)		216 (47)	242 (49)	
Pharmacists	10 (10)	9 (6)		77 (17)	80 (16)	
Others	13 (13)	21 (13)		89 (19)	91 (19)	
NA	2 (2)	3 (2)		3 (1)	1(1)	
*Demographical characteristics*						
Age (years), median (25–75° perc)	7 (2–14)	3 (1–10)	<0.05	52 (40–57)	75 (69–81)	
Sex, n (%)						
Males	49 (50)	91 (56)		263 (57)	276 (56)	
Females	48 (50)	70 (44)	0.42	197 (43)	214 (44)	0.48
*Antimicrobial therapy*						
Treatment duration (days), median (25–75° perc)	13 (9–26)	2 (1–4)	<0.05	18 (11–25)	22 (12–34)	<0.05
TTO (days), median (25–75° perc)	12 (9–25)	1 (1–3)	<0.05	14 (10–22)	19 (11–27)	<0.05
Treatment discontinuation, n (%)						
At the occurrence of the AE (±1 day)	70 (72)	139 (86)		223 (51)	267 (55)	
After the onset of the AE (>1 day)	27 (28)	22 (14)	<0.05	227 (49)	223 (45)	<0.05
*Reported antimicrobial therapy* *in the reports, n (%)*						
J01A (tetracyclines)	3 (3)	0 (0)		12 (3)	19 (4)	0.54
J01B (amphenicols)	0 (0)	3 (2)		0 (0)	1 (1)	
J01C (beta-lactam antibacterials, penicillins)	64 (66)	109 (68)	0.88	290 (63)	309 (63)	0.85
J01D (other beta-lactam antibacterials)	48 (50)	52 (32)	<0.05	209 (45)	200 (41)	0.17
J01E (sulfonamides and trimethoprim)	18 (19)	3 (2)	<0.05	69 (15)	78 (16)	0.68
J01F (macrolides, lincosamides and streptogramins)	17 (18)	30 (19)	0.95	83 (18)	67 (14)	0.17
J01G (aminoglycoside antibacterials)	17 (18)	5 (3)	<0.05	30 (7)	52 (11)	<0.05
J01M (quinolone antibacterials)	4 (4)	0 (0)		94 (20)	94 (19)	<0.05
JO1X (colistine, metronidazole, linezolid)	7 (7)	4 (3)	0.11 ^#^	41 (9)	34 (7)	0.51
J01XA01/J01AX02 (Vancomycin/Teicoplanin)	35 (36)	12 (7)	<0.05	159 (35)	184 (38)	0.63
More than one penicillin (J01C)	17 (18)	20 (12)	0.34	132 (29)	133 (27)	0.07
More than one other beta-lactam (J01D)	13 (13)	4 (3)	<0.05	55 (12)	43 (9)	0.18
More than one drug class of interest (J01C/J01D/J01XA01/J01AX02)	65 (67)	62 (39)	<0.05	345 (75)	389 (79)	<0.05

AE: adverse event; FAERS: FDA Adverse Event Reporting System; NA: not available; TTO: time to onset. * Mann–Whitney U test for continuous variables and Chi-square test for categorical variables when comparing delayed TTO vs. early TTO. ^§^ Kruskal–Wallis test for continuous variables and Chi-square test for categorical variables when comparing age groups for delayed TTO. ^#^ Fisher’s exact test.

## Data Availability

The datasets analyzed during the current study are available in the public domain: https://fis.fda.gov/extensions/FPD-QDE-FAERS/FPD-QDE-FAERS.html (accessed on 17 October 2024).

## Supplementary Materials

The following supporting information can be downloaded at: https://www.mdpi.com/article/10.3390/antibiotics15080749/s1, Supplementary Material S1; Figure S1; Figure S2.

## Abbreviations

The following abbreviations are used in this manuscript:

CADRs	Cutaneous adverse drug reactions
SCARs	Severe cutaneous adverse reactions
FAERS	FDA Adverse Event Reporting System
ADRs	Adverse drug reactions
RWD	Real-world data
RWE	Real-world evidence
TTO	Time to onset
SJS	Stevens–Johnson syndrome
TEN	Toxic epidermal necrolysis
ICSRs	Individual case safety reports
