# Supplementary material for: Real-World Patterns of Delayed Cutaneous Adverse Reactions to Antimicrobial Therapy in Children: A Case Series and FAERS Analysis Across Age Groups
Source: Antibiotics (Basel). 2026 Aug 3;15(8):749. doi: 10.3390/antibiotics15080749 (PMC13509894; doi:10.3390/antibiotics15080749)

Supplementary Material S1. list of clinically relevant MedDRA terms

RASH MACULO-PAPULAR
RASH MACULAR
RASH MACULOVESICULAR
RASH MORBILLIFORM
HARLEQUIN SKIN REACTION
RASH ERYTHEMATOUS
RASH PAPULAR
RASH SCARLATINIFORM
EXFOLIATIVE RASH
RASH VESICULAR
EYELID RASH
GENITAL RASH
VASCULITIC RASH
MUCOCUTANEOUS RASH
VULVOVAGINAL RASH
RASH FOLLICULAR
RASH RUBELLIFORM
RASH PAPULOSQUAMOUS
PERINEAL RASH

Supplementary Figure 1. Flowchart for retrieving the cases of interest

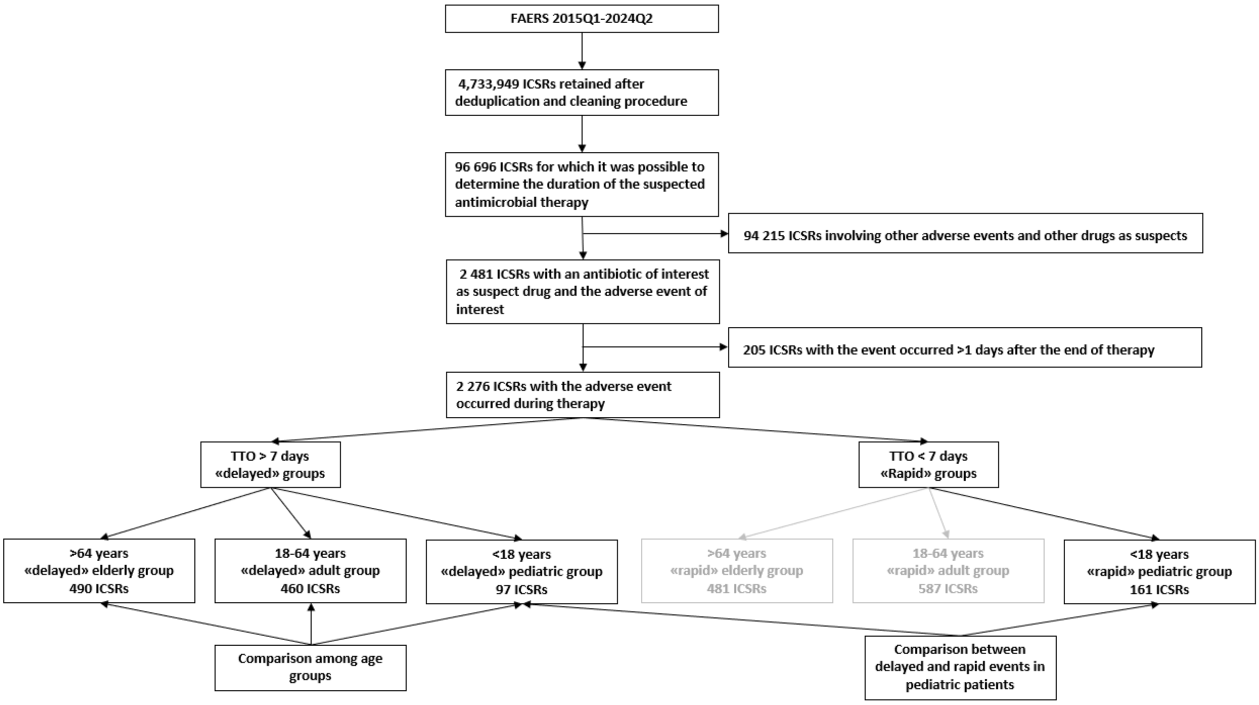

Supplementary Figure 2. Word cloud depicting indication therapies related to infections (when available): A) Adults experiencing delayed adverse events; B) Children experiencing delayed adverse events; C) Children experiencing rapid-onset adverse events; D) Elderly patients experiencing delayed adverse events.

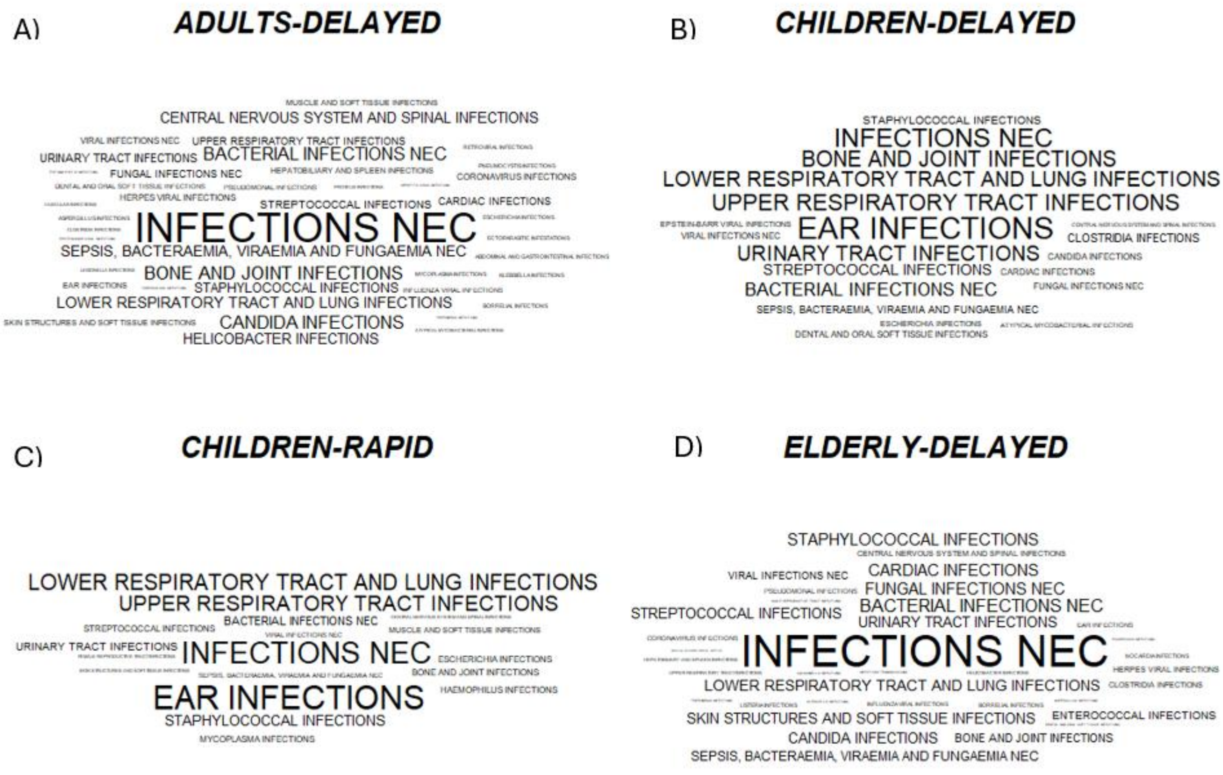

Supplement: Supplementary file 1 [file antibiotics-15-00749-s001.zip › antibiotics-4390959-supplementary.pdf]
